# A novel strategy to study apomixis, automixis, and autogamy in plants

**DOI:** 10.1007/s00497-024-00499-6

**Published:** 2024-03-02

**Authors:** Petra Šarhanová, Ľuboš Majeský, Michal Sochor

**Affiliations:** 1 Department of Botany and Zoology, Masaryk University, Kotlářská 267/2, 611 37 Brno, Czech Republic; 2 Faculty of Science, Department of Botany, Palacký University in Olomouc, Šlechtitelů 27, 783 71 Olomouc-Holice, Czech Republic; 3 Centre of the Region Haná for Biotechnological and Agricultural Research, Crop Research Institute, Šlechtitelů 29, 78371 Olomouc, Czech Republic

**Keywords:** Apomixis, Automixis, SSR-seq, FCSS, *Rubus*, *Taraxacum*

## Abstract

**Key message:**

The combination of a flow cytometric seed screen and genotyping of each single seed offers a cost-effective approach to detecting complex reproductive pathways in flowering plants.

**Abstract:**

Reproduction may be seen as one of the driving forces of evolution. Flow cytometric seed screen and genotyping of parents and progeny are commonly employed techniques to discern various modes of reproduction in flowering plants. Nevertheless, both methods possess limitations constraining their individual capacity to investigate reproductive modes thoroughly. We implemented both methods in a novel manner to analyse reproduction pathways using a carefully selected material of parental individuals and their seed progeny. The significant advantage of this approach lies in its ability to apply both methods to a single seed. The introduced methodology provides valuable insights into discerning the levels of apomixis, sexuality, and selfing in complex *Rubus* taxa. The results may be explained by the occurrence of automixis in *Rubus*, which warrants further investigation. The approach showcased its effectiveness in a different apomictic system, specifically in *Taraxacum*. Our study presents a comprehensive methodological approach for determining the mode of reproduction where flow cytometry loses its potential. It provides a reliable and cost-effective method with significant potential in biosystematics, population genetics, and crop breeding.

**Supplementary Information:**

The online version contains supplementary material available at 10.1007/s00497-024-00499-6.

## Introduction

Apomixis, the asexual reproduction via seeds, has garnered scientific attention for decades. This mode of reproduction enables the preservation of the mother plant's genotype in offspring, potentially influencing evolution differently than sexual reproduction. Concomitant benefits include the fixation of successful genotypes (e.g., Maynard-Smith 1978; Sailer et al. 2016; Liu et al. 2023) and elevated heterozygosity associated with hybrid origin and polyploidy (e.g., Gornal, 1999; Richards 2003; Paun et al. 2006). On the other hand, apomicts may also face challenges such as mutation accumulation (Muller's ratchet; Muller 1964) and a lower capacity to adapt to changing environments (Stebbins 1957; Maynard-Smith 1978). Most apomicts retain the capacity for sexual reproduction, which can facilitate effective escape from mutation accumulation and enhance population dynamics (Hojsgaard and Hörandl 2015; Hodač et al. 2019).

**Fig. 1:**
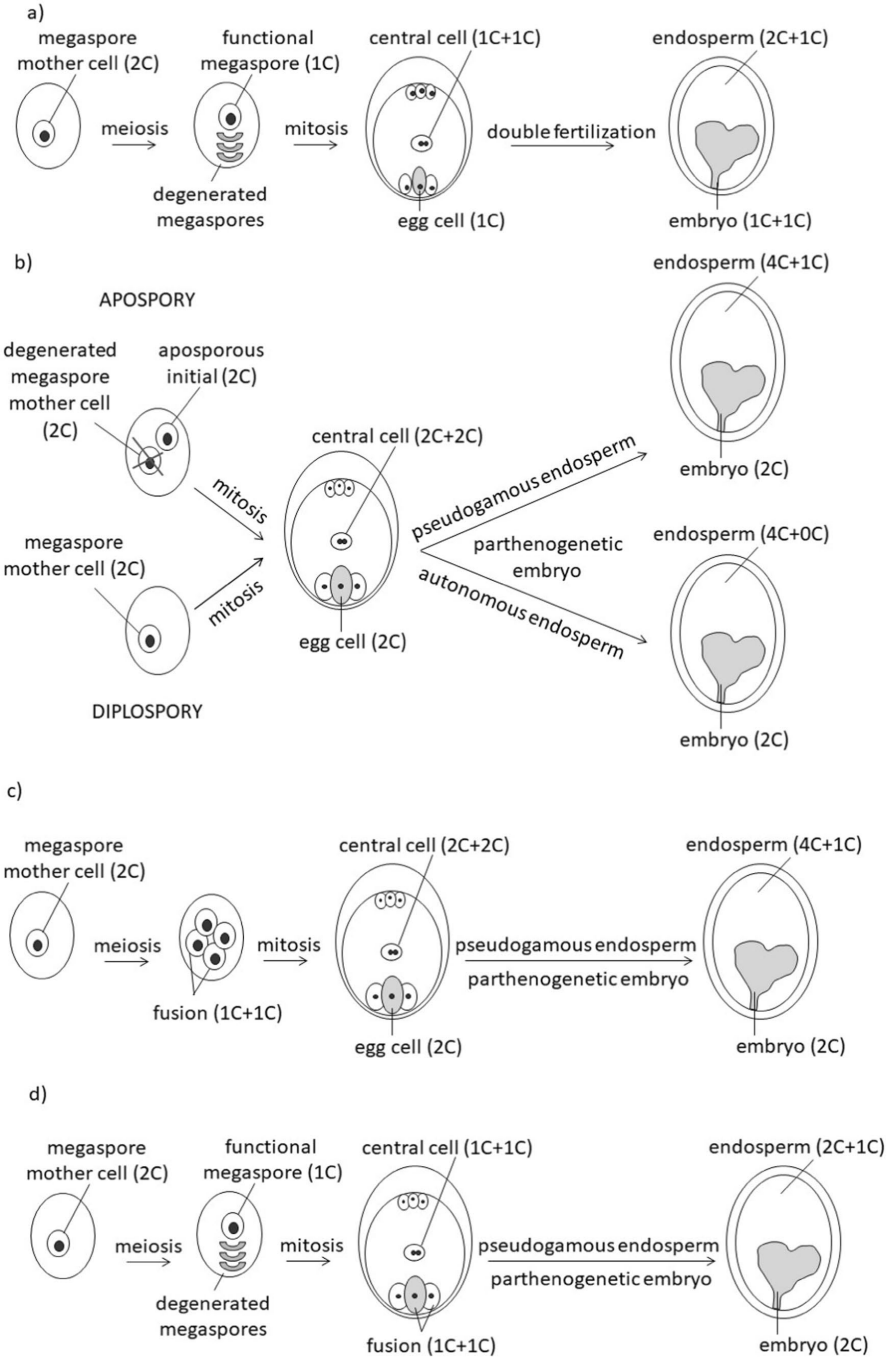
Mechanisms of seed development and ploidy levels of each reproductive cell: **a** sexuality, **b** gametophytic apomixis – apospory and diplospory, **c** automixis type I, **d** automixis type II (particular details may differ in different taxa). “C” refers to the holoploid genome sensu Greilhuber et al. (2005); the first “C” defines the maternal and the second paternal genome, if present

Various types of asexual seed reproduction encompass gametophytic modes of apomixis, such as apospory (e.g., *Rubus*) and diplospory (e.g., *Taraxacum*), distinguished by the specific cell type responsible for initiating the formation of the embryo sac (as depicted in Fig. 1b). Additional type of asexual seed production is sporophytic apomixis, commonly referred to as adventitious embryony (e.g., *Citrus*) (e.g., Gustafsson 1946).

Determining the reproductive mode is the key to evaluating and interpreting the apomicts' evolutionary success. A rapid method for confirming the mode of reproduction in angiosperms with *Polygonum*-type embryo sacs (ES) is Flow Cytometric Seed Screen (FCSS; Matzk et al. 2000). This method allows for extrapolating the origin of the embryo (parthenogenetic or fertilized) and ES (reduced or unreduced) by comparison of relative genome sizes of embryo and endosperm. In sexual reproduction, this ratio is 2C:3C (Fig. 1a), where C is defined as the 'holoploid genome' (sensu Greilhuber et al. 2005). In contrast, meiosis is omitted during apomixis, and the embryo develops parthenogenetically within an unreduced ES. The endosperm forms without fertilization of the central cell (i.e., autonomously), resulting in an embryo:endosperm ratio of 2C:4C, or after fertilization (i.e., pseudogamy), usually resulting in a ratio of 2C:5C (Fig. 1b).

Moreover, certain apomictic species exhibit considerable variability in the ploidy level of the endosperm, which implies either a fusion of a variable number of nuclei in the embryo sac and/or a variable number or ploidy level of sperm cells (Pratt and Einsett, 1955; Šarhanová et al. 2012; Dobeš et al. 2013). As a result, FCSS does not always provide an accurate reflection of the actual origin of the seed. Despite this limitation, FCSS results are widely accepted and serve as a basis for drawing biosystematic implications.

Another reproductive mode that merits consideration in plant evolution is automixis (Mogie 1986). It is the primary type of gamete fusion in parthenogenetic animals (Simon et al. 2003). However, in the case of angiosperms, it is only assumed to exist (e.g., Gerlach 1965; Antonius and Nybom 1995) and has never been rigorously experimentally confirmed. During automixis, meiosis takes place, but instead of regular fertilization, the ploidy of the embryo is reconstituted by the duplication or fusion of two reduced nuclei, where both are the products of a single meiotically dividing cell. In theory, two types of such fusion might occur at different time points. First, after the generation of meiotically reduced megaspores, two of them fuse and give origin to an unreduced embryo sac (hereafter termed type I; Fig. 1c). Second, in the reduced embryo sac, the egg cell fuses with some other reduced nuclei (hereafter termed type II; Fig. 1d). In both cases, automixis can result in reduced heterozygosity, with the extent depending on the type of automixis, number of crossovers, or type of fusion (terminal or central; Nougué et al. 2015). Notably, the automixis maintains the DNA repair function of meiosis, and in automictic animals, selection for preserving meiosis is stronger than maintaining a high level of heterozygosity (Mirzaghaderi and Hörandl 2016). The existence of automixis and possible evolutionary forces driving the preservation of meiosis in plants, particularly in allopolyploids characterized by higher levels of heterozygosity, still await scientific elucidation. The genus *Rubus* encompasses a diverse range of reproductive modes, including apomixis (Šarhanová et al. 2012) and suggested automixis (Gerlach 1965; Antonius and Nybom 1995). Thus, blackberries were selected as the primary plant taxa to investigate and provide experimental evidence of automixis in angiosperms.

Simple sequence repeat genotyping by sequencing (SSR-seq) is an amplicon sequencing technique (Šarhanová et al. 2018), enabling simultaneous genotyping of multiple loci in terms of length and sequence, thereby increasing the detected variability of each locus. The presented methodological approach innovatively combines the benefits of FCSS and genotyping via SSR-seq, employing both methods in every individual seed. To assess the performance and efficacy of this integrated approach, we selected two distinct plant genera characterized by divergent apomictic reproductive modes.

i. *Rubus* subgenus *Rubus* (Rosaceae), a highly variable taxon of thorny shrubs exhibiting prevalent polyploidy but rare diploid occurrence (only three diploid species in Europe). The diploids reproduce solely sexually, while the odd-polyploids reproduce exclusively by pseudogamous apospory (Fig. 1b). The most common tetraploids exhibit varying degrees of residual sexuality, resulting in a high hybridization rate and offspring of diverse ploidy levels. Male meiosis may also be affected, resulting in decreased viability of pollen. Nonetheless, viable pollen is meiotically reduced (Gustafsson 1943). In sexually developing and most apomictic seeds, the endosperm arises from the fusion of two polar nuclei and a single sperm cell. In some cases, however, a fusion of additional sperm cells or maternal nuclei of the embryo sac can fuse, resulting in elevated ploidy levels of endosperm (Pratt and Einsett, 1955; Šarhanová et al. 2012).
ii. *Taraxacum* (Asteraceae), a cosmopolitan genus of perennial herbs, comprises diploid and polyploid taxa. Diploid species reproduce strictly sexually, while polyploids are obligately apomictic. The polyploid taxa contribute to 90% of species richness, and the diploid taxa account for the remaining 10% of species richness within the genus (reviewed in Majeský et al. 2017). The type of gametophytic apomixis utilized by polyploid dandelions is meiotic diplospory (Fig. 1b). Seed progeny formation is entirely independent of the male gametophyte, and the embryo develops parthenogenetically from an unreduced female megaspore. At the same time, endosperm formation is autonomous (without the participation of male gametes in fusion with central cell; Gustafsson 1946; Tas and van Dijk 1999).

Our main objectives were to (i) develop a rapid, cost-effective, and reliable approach for the genotyping of seeds, particularly in scenarios where both the quantity of seed material and available DNA may be limited; (ii) determine the mode of reproduction by FCSS and validate the results by employing molecular markers on the same seed for both analyses; (iii) evaluate the potential of the method for the detection of automixis in plants using the model genus *Rubus*; and (iv) evaluate the method in another apomictic complex, *Taraxacum*. The presented approach allows for precise determination of the reproductive mode across diverse taxa, and its potential benefits extend to clonality studies, population dynamics research, or applications in agriculture, such as marker-assisted breeding.

## Materials and methods

### Plant material

*Taraxacum*: two diploid sexual autogamous species (*T. gilliesii* Hook. and Arn. and *T. cygnorum* Hand.-Mazz.) and two triploid obligate apomictic species (*T. cristatum* Kirschner et al*.*, and *T. pudicum* Vašut et Majeský) were selected from the experimental greenhouse at the Department of Botany, Palacky University in Olomouc (Table 1, TRX set). Seeds were harvested from isolated, and in the case of apomicts, emasculated inflorescences to prevent accidental hybridization and verify the mode of reproduction.

**Table 1:** List of specimens used in the experiment

Set	Species	ID	Locality	Ploidy	Reproduction mode
TRX	*T. gilliesii*	GILL	AR, Tierra del Fuego, Est. Haberton	2x	Sexual (autogamous)
	*T. cygnorum*	CYG	AU, south‒west Victoria	2x	Sexual (autogamous)
	*T. pudicum*	PUD 25	CZ, Budišov	3x	Apomictic
	*T. cristatum*	GA5	AT, Gänsendorf	3x	Apomictic
RUB*ex*	*R. ulmifolius*	RJV5	IT, Acquapendente	2x	Sexual
	*R. bifrons*	R150-20	CZ, Horažďovice	4x	Facult. apomictic
	*R. epipsilos*	R11-10	CZ, Mříč	4x	Facult. apomictic
	*R. vatavensis*	R127-4	CZ, Lhenice	4x	Facult. apomictic
	*R. vatavensis*	R127-12	CZ, Lhenice	4x	Facult. apomictic
	*R.* ser. *Glandulosi*	R143-9	CZ, Prachatice	4x	Apomictic
RUB*nat*	*R.* ser. *Glandulosi*	Kórnik	PL, Borówiec	4x	Facult. apomictic
	*R.* ser. *Glandulosi*	MS137/20	CZ, the Luž Mt	4x	Sexual
	*R.* ser. *Glandulosi*	MS165/20	PL, Wólka Małkowa	4x	Facult. apomictic
	*R. apricus*	MS176/20	PL, Lasy Janowskie	4x	Facult. apomictic
	*R.* ser. *Glandulosi*	Ms196/20	CZ, Ramzová	4x	Sexual

TRX – maternal individuals and seed progeny of *Taraxacum* species, RUB*ex* – maternal and paternal individuals and seed progeny of *Rubus* from experimental crosses, RUB*nat* – maternal individuals and seed progeny of *Rubus* collected in nature

*Rubus* subgenus *Rubus*: one individual representing a diploid sexual species (*R. ulmifolius* Schott, ser. *Discolores*) and five tetraploid individuals (one from the series *Glandulosi* without species recognition, one *R. bifrons* Vest, ser. *Discolores*, one *R. epipsilos* Focke, ser. *Radula*, and two individuals, *R. vatavensis* Žíla et Trávn., ser. *Radula*) with a variable level of apomixis/sexuality (Šarhanová et al. 2012) were selected from the cultivation of Masaryk University, Brno, to perform crossing experiments (Table 1, RUB*ex* set). Additionally, five individuals and their seed progeny (four individuals of *R.* ser. *Glandulosi* and one of *R. apricus* Wimm., ser. *Hystrix*) collected in their natural habitats were included (Table 1, RUB*nat* set).

### Crossing experiment of *Rubus*

A series of controlled pollination experiments involved one diploid and five tetraploid individuals of *Rubus* (Table 1, RUB*ex* set; Table 2). Four pollination treatments were performed: (i) self-pollination within a single individual, (ii) cross-pollination between two different species, (iii) simulation of open-pollination with a pollen mixture from 2 to 4 species, and (iv) nonpollination control to assess the capacity for autonomous endosperm development. The flower buds of selected individuals were emasculated prior to blooming and covered with fabric bags. The anthers were collected in Eppendorf tubes for pollen dusting. One day after emasculation, the stigma was examined for receptivity (glossy appearance and spacing of styles), and collected pollen was directly brushed onto the stigma of the recipient using a brush. The flower was again covered with a fabric bag until fruit maturation. The seeds were harvested, cleaned, dried, and stored under cold conditions at 4 °C until FCSS analyses.

**Table 2:** List of crossing combinations of *Rubus* species (RUB*ex* set)

Species	Maternal individual ID	Pollen donor ID	Crossing experiment ID	Pollination treatment	Number of developed seeds	Number of seeds for FCSS	Abortion rate among the seeds for FCSS (%)	Number of SSR-analysed seeds
*R. ulmifolius*	RJV5	R11-10	21–515	ii	18	10	100.0	0
*R. ulmifolius*	RJV5	R127-4	21–519	ii	11	10	100.0	0
*R. ulmifolius*	RJV5	R143-9	21–514	ii	10	10	100.0	0
*R. bifrons*	R150-20	R150-20	21–156	i	28	10	20.0	3
*R. bifrons*	R150-20	RJV5	21–378	ii	15	10	20.0	0
*R. bifrons*	R150-20	R11-10	21–116	ii	18	10	10.0	6
*R. bifrons*	R150-20	R127-4	21–239	ii	24	10	30.0	4
*R. bifrons*	R150-20	R143-9	21–238	ii	17	10	20.0	4
*R. bifrons*	R150-20	ALL	21–357	iii	13	12	66.7	2
*R. epipsilos*	R11-10	R11-10	21–25	i	19	7	0.0	5
*R. epipsilos*	R11-10	RJV5	21–485	ii	16	10	50.0	3
*R. epipsilos*	R11-10	R127-4	21–283	ii	13	10	30.0	4
*R. epipsilos*	R11-10	R143-9	21–205	ii	18	8	0.0	4
*R. epipsilos*	R11-10	R150-20	22–42	ii	24	8	25.0	0
*R. epipsilos*	R11-10	RJV5 + R11-10	21–439	iii	26	19	63.2	3
*R. epipsilos*	R11-10	RJV5 + R127-4	21–440	iii	9	9	55.6	2
*R. epipsilos*	R11-10	ALL	22–104	iii	12	12	25.0	0
*R. vatavensis*	R127-12	R127-12	21–54	i	15	15	73.3	2
*R. vatavensis*	R127-12	RJV5	20–92	ii	17	17	82.4	0
*R. vatavensis*	R127-12	R11-10	21–80	ii	14	6	16.7	5
*R. vatavensis*	R127-4	R143-9	21–383	ii	43	5	0.0	3
*R. vatavensis*	R127-4	R143-9	21–48	ii	21	6	0.0	2
*R. vatavensis*	R127-12	R150-20	21–77	ii	24	10	0.0	0
*R. vatavensis*	R127-4	ALL	22–126	iii	16	10	40.0	0
*R*. ser. *Glandulosi*	R143-9	R143-9	21–37	i	11	10	40.0	2
*R*. ser. *Glandulosi*	R143-9	R11-10	22–15	ii	9	5	40.0	0
*R*. ser. *Glandulosi*	R143-9	R127-4	21–217	ii	8	8	50.0	3
*R*. ser. *Glandulosi*	R143-9	R150-20	21–326	ii	10	10	80.0	0
*R*. ser. *Glandulosi*	R143-9	RJV5 + R127-4	21–508	iii	15	10	40.0	5
*R*. ser. *Glandulosi*	R143-9	ALL	21–352	iii	14	10	30.0	3

Each line describes the crossing experiment of a single flower resulting in an aggregate fruit, the number of developed seeds per fruit, the number of seeds used for FCSS analyses with abortion rate, and the number of seeds genotyped with SSR-seq. i – self-pollination, ii – cross-pollination with single species, iii – cross-pollination with 2–4 species. For the ID of each individual, see Table 1

### Flow cytometry

Absolute genome sizes were estimated by flow cytometry using a Partec CyFlow ML instrument (Partec GmbH, Münster, Germany). First, the leaf tissue of the parental plant and internal standard *Lycopersicon esculentum* ('Stupické polní rané', 2C = 1.96 pg; Doležel et al. 1989) were chopped together in 1 ml of Galbraith buffer (Galbraith et al. 1983) with few modifications (45 mM MgCl_2_, 20 mM MOPS, 30 mM sodium citrate, 0.1% Triton X-100, 1% PVP). The solution was stained with 50 µl propidium iodide (final concentration 50 mg ml^−1^). While the genome size variability between different species and series is comparatively lower than the variation observed between ploidy levels (Krahulcová et al. 2013; Sochor et al. 2019), the absolute genome size served for the ploidy level determination of each parental individual and was calculated from the peak positions of the sample and the standard.

**Fig. 2:**
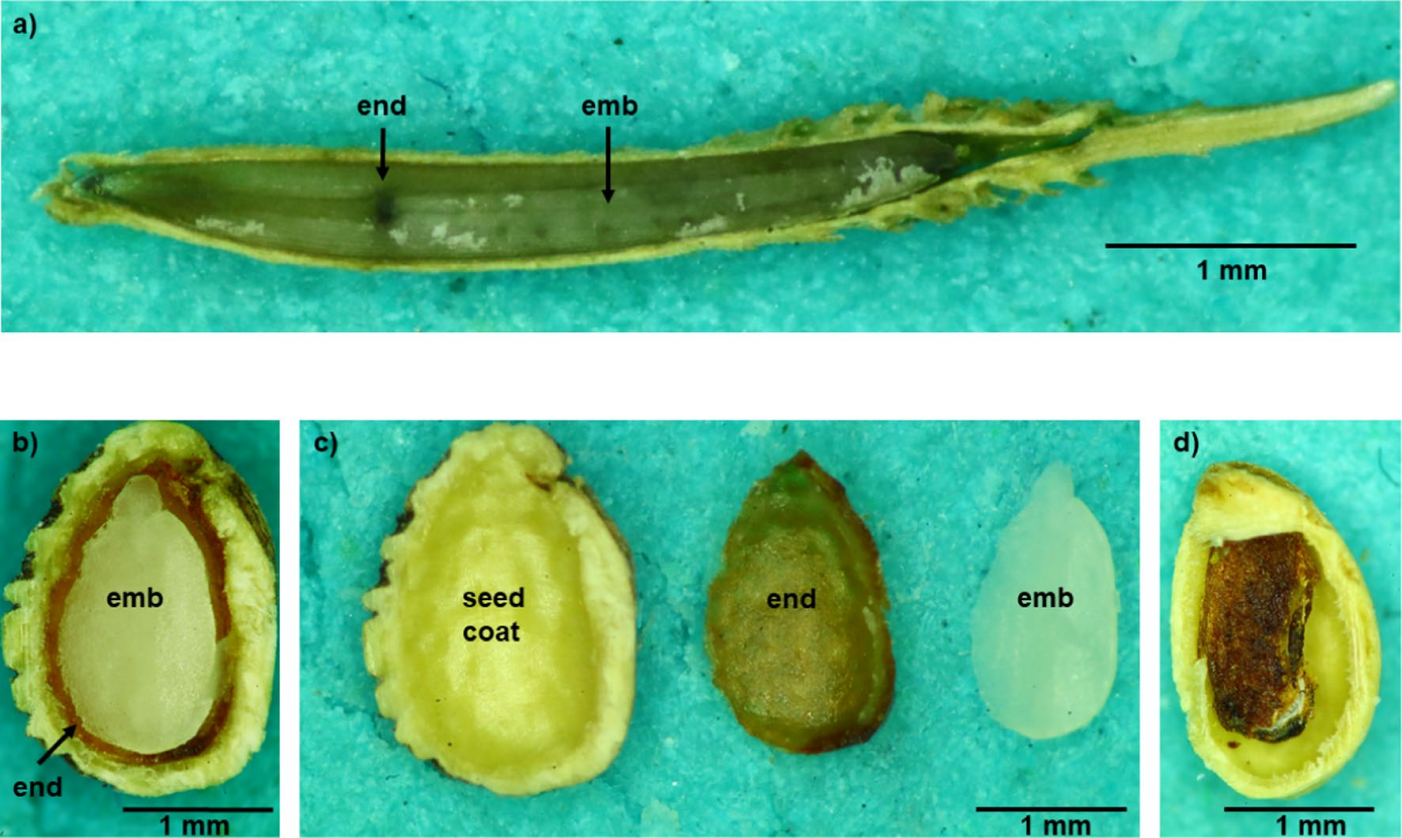
Sections of fully developed seeds of **a** *Taraxacum* and **b**–**d** *Rubus.* **c** embryo and endosperm separated from the seed coat and **d** aborted seed. emb – embryo, end – endosperm

Determination of ploidy level of the embryo and endosperm was performed using the same instrument, following Šarhanová et al. (2012). The ploidy levels of the embryo and endosperm served for reproduction mode determination based on the rationale in Fig. 1, using only half of each seed (Fig. 2). The second half of the lengthwise sectioned seed was preserved in an Eppendorf tube at 4 °C for subsequent DNA extraction and SSR-seq. The number of aborted seeds (shrunken and dark-colored) per individual and the number of seeds selected for molecular analyses were recorded (Table 2).

### DNA extraction

The DNA of parental individuals was extracted from silica gel-dried leaves with a Spin Plant Mini Kit (Invisorb) and diluted to a concentration of 10 ng/µl. The DNA from the second half of the seeds (the first half was used for FCSS) was extracted following the protocol of Brewster and Paoli (2013) with slight modifications. The seed tissue was shredded in a 2 ml Eppendorf tube with metal beads or a 1.5 ml tube with sand and pestle; 50 µl of HotShot buffer (125 mM NaOH, 1 mM EDTA, 0.1% Tween 20) was added, vortexed, and incubated at 65 °C for 30 min. Subsequently, 50 µl of neutralizing solution (125 mM HCl, 10 mM Tris–HCl) was added, vortexed, and refrigerated for 1 h to allow sedimentation. The resulting DNA extract was diluted at a ratio of 1:10 with ddH_2_O to obtain the working solution for PCR.

### PCR and SSR-seq

For the sequencing of microsatellite markers, a variable number of loci were tested: six for *Taraxacum* (Supporting Information Table S1) and twelve for *Rubus*. The tested loci of *Rubus* belonged to seven linkage groups reflecting the basic chromosome number of the genus (Woodhead et al. 2008, Supporting Information Table S2). The linkage of *Taraxacum* loci is not known and cannot be excluded. PCR amplification of each locus was performed using a Multiplex PCR Kit (Qiagen) following Standard Multiplex PCR from the manufacturer's Handbook (37 cycles, 62 °C annealing temperature, and 0.2 µM primer concentration). Five loci of *Taraxacum* and nine loci of *Rubus* were successfully amplified in maternal individuals and were used for the initial sequencing test with four parental individuals and four seeds (one from each parent). PCR amplification outputs and locus variability were evaluated. Three loci for *Taraxacum* and seven for *Rubus* showed sequence variability, providing high-quality results in all tested parental individuals and seed progeny.

The final set of seven *Rubus* loci belonged to five linkage groups (Supporting Information Table S2). Only loci 1B06 and 72H02 were assigned to the same group, with a calculated distance of 103.2 centimorgans (Woodhead et al. 2008). Consequently, it is anticipated that these loci will likely undergo separation through recombination during meiosis. The linkage group of locus RhM023 remains unidentified, and its potential linkage to other markers cannot be ruled out. For the seven loci of *Rubus*, forward and reverse primers were ordered with 8-bp appended barcodes on the 5’-ends, enabling higher multiplexing within the sequencing library (Šarhanová et al. 2018). The final PCR was performed in two multiplex reactions for each individual/seed of *Rubus* (80 samples) and a single reaction for *Taraxacum* (8 samples), adjusting primer concentrations based on initial sequencing outputs (Supporting Information Table S3). The PCR was performed twice for several seeds to detect possible genotyping errors.

All PCR products were combined to create a sequencing library consisting of ten *Rubus* samples, each labelled with a unique barcode, and one *Taraxacum* sample. The volume of each sample in the pool was determined based on the number of loci, sequencing outputs from the initial test, and the ploidy level of the parental individual or embryo. Pooled PCR products were purified using 1.2 × SPRIselect beads (Beckman Coulter). Sequencing libraries were created using the Swift 2S® Sonic Flexible DNA Library Kit (Swift Biosciences) and TruSeq DNA Unique Dual Indexes (Illumina), following the manufacturer's protocols with halved reagent volumes. Detailed information on all tested loci, including their length, repetitive motif, and primers, can be found in Supporting Information Tables S1 and S2. Paired-end sequencing was performed at the CEITEC facility (Brno, Czech Republic) on the NextSeq platform with mid-output and 300 cycles (Illumina), using only part of the sequencing capacity to generate approximately 10,000 paired-end reads per locus and individual.

### Data analyses

The data analysis pipeline was created in Geneious Prime 2021.2.2 (https://www.geneious.com). The sequences of each library were trimmed for quality (below 6) and length (less than 100 bp) using BBDuk. Paired reads were merged, and barcoded forward and reverse primers were used to separate the reads, allowing a single mismatch. Complementary forward and reverse sequences were grouped, and each group represented a single locus in a single individual. De novo assembly was performed with a custom sensitivity setting, allowing for 1% mismatches and one ambiguity per read.

Sequence variations such as SNPs, indels, and the number of SSR motifs were considered to characterize the genotype of each parental individual. The first twenty contigs from de novo assembly were saved, and consensus sequences, including coverage information, were generated, each representing an allele. Contigs containing mixed primer-attached barcodes were excluded from the analysis. A threshold based on read coverages was used to differentiate true alleles from PCR/sequencing errors (Supporting Information Fig. S1). The identified true alleles in maternal individuals of *Taraxacum* and *Rubus* served as references for progeny genotyping (Supporting Information Figs. S2 and S3).

The analysis was then performed for each progeny. The resulting twenty contigs were aligned with the reference alleles. The coverage of recorded alleles was used to determine allelic dosage in polyploids (e.g., A1A1A2A3 vs. A1A2A2A3 vs. A1A2A3A3) and potentially detect paternal alleles in the endosperm of the RUB*ex* set.

Based on the nature of the experimental plant material, three datasets were created to compare the embryo's SSR genotype with the maternal/paternal SSR genotype: (i) TRX set – included the maternal individuals and seed progeny of *Taraxacum* species; ii) RUB*nat* set – included maternal individuals and seed progeny of *Rubus* individuals collected in nature; iii) RUB*ex* set – included maternal and paternal individuals and seed progeny of experimental crosses. The evaluation of the reproductive mode slightly differed for each dataset due to differences in the availability of paternal genotypes. When only the maternal genotype was known (TRX set and RUB*nat* set), the genotypes of the sexually originated embryos differed from their mother plants due to i) the presence of novel paternal alleles (outcrossing), (ii) the absence of some maternal alleles, and/or (iii) different dosages of maternal alleles without the presence of novel alleles (selfing). In the case of apomixis, the progeny genotype was expected to be identical to its seed parent, with the possibility of detecting somatic mutations. Following these presumptions, seeds were considered to have arisen via apomixis if (i) the genotype of the embryo possessed an identical genotype to its mother plant, (ii) the changed dosage from the expected SSR genotype occurred in a maximum of one locus, or (iii) there was a single nucleotide mutation in a maximum of one allele compared to the maternal genotype. Progeny from the out-crossing experiment (RUB*ex* set, treatments ii and (iii) was classified as having arisen through sexual processes when the SSR genotype of the embryo represented a mixture of alleles from both parental genotypes. However, if the progeny showed changes in allelic dosage for multiple loci and/or lacked maternal alleles for multiple loci, in the simultaneous absence of any novel (paternal) alleles, they were considered to arise via automixis (FCSS ratio depending on the type of automixis, see Fig. 1 and Table 3).

**Table 3:** Characterization of a possible origin of embryo based on SSR genotype compared to maternal/paternal genotypes and FCSS embryo:endosperm ratio

Embryo origin	SSR-seq	SSR-seq	FCSS
	♀ alleles in embryo	♂ alleles in embryo	Embryo:endosperm
APO	All present, no dosage change	None	2C:4C or 2C:5C or 2C:6C*
SEX^out^	½ present	½ present	2C:3C
SEX^self^	Partly absent and/or changed dosage	Same as ♀	2C:3C
AUT-I	Partly absent and/or changed dosage	None	2C:4C or 2C:5C or 2C:6C*
AUT-II	Partly absent and/or changed dosage	None	2C:3C
PH	½ present	None	1C:3C
B_III_	all present	½ present	3C:5C

APO – apomictic, SEX^out^ – sexual out-crossing, SEX^self^ – sexual selfing, AUT-I – automixis type I, AUT-II – automixis type II (for the explanation of automixis see Fig. 1), PH – polyhaploid, B_III_ – hybrid with elevated ploidy. * depending on paternal contribution with none, one or two sperm cells

Based on the criteria mentioned above, the following reproductive pathways were determined for each analysed seed of *Rubus*: APO (apomictic), SEX^out^ (sexual – out-crossing), SEX^self^ (sexual – selfing), AUT-I (automictic type I), AUT-II (automictic type II), PH (polyhaploid), and B_III_ (hybrid with elevated ploidy) (Table 3). The representation of each category in each maternal individual was calculated.

The loci were tested for capacity to correctly determine the offspring's parentage in Polygene v1.6 (Huang et al. 2020). The sexually originated seeds from the RUB*ex* set (FCSS ratio 2C:3C, ploidy 4x) were analysed based on every single locus and on combinations of 2–7 loci. The category was set to “identifying the father when the mother is known” applying the likelihood method (Marshall et al. 1998), allowing for selfing and running 100,000 simulations. The method can find the optimal parent even if some parents cannot be excluded based on two hypotheses: the alleged parent is or is not the true parent. Each alleged parent is assigned an LOD score (the natural logarithm of the ratio of these two likelihoods), and the individual with the highest positive LOD score is considered the true paternal individual.

## Results

### Flow cytometric seed screen

In all seeds with visually developed embryos and endosperms (Fig. 2), flow cytometry successfully determined the ploidy level of both parts, which served as a proxy for reproduction mode determination. Examples of the variable FCSS outputs are provided in Supporting Information Fig. S4. Empty seeds and those with degenerated inner tissues were considered aborted and not used for further analyses (Fig. 2d). In the genus *Taraxacum*, the FCSS method confirmed the expected sexual reproduction of diploid taxa (embryo:endosperm ratio 2C:3C) and obligate apomixis of the investigated triploid taxa (2C:4C; Supporting Information Table S4).

The scenario was more complex in the genus *Rubus*. The attempted crossings for autonomous endosperm development within the RUB*ex* set yielded no seeds. Additionally, all seed progeny from the diploid individual *R. ulmifolius* (RJV5) were aborted. Among the tetraploid taxa, a variable level of aborted seeds (Table 2), reduced/unreduced embryo sacs, and fertilized/parthenogenetic embryos was observed (Supporting Information Table S5), causing additional variability in the embryo ploidy level. Analyses of seeds from the RUB*nat* and RUB*ex* sets indicated reduced ploidy levels in nine embryos, suggesting parthenogenetic development of reduced egg cells (dihaploids in this case). Conversely, peaks corresponding to hexaploid embryos were detected in ten seeds, indicating the fertilization of unreduced egg cells and the formation of B_III_ hybrids. The endosperm ploidy level varied from triploid (3x) to quindecimploid (15x), reflecting the ploidy of both parents and the number of maternal nuclei and sperms contributing to endosperm development.

Interesting results emerged in three flowers subjected to open pollination with a mixture of pollen from diploid and tetraploid taxa (RUB*ex* set, experiment IDs: 21–439, 21–440, 21–508; Supporting Information Table S6). Based on FCSS analysis of 10 seeds, the tetraploid pollen donor took part in the origin of seven seeds, the diploid pollen donor took part in two seeds, and in one seed, FCSS suggested a heteroploid origin of the endosperm (embryo:endosperm = 4C:11C, seed ID: 21–440-4;), indicating polytubey during fertilization.

### SSR-seq

Four maternal *Taraxacum* individuals and their 27 seeds were SSR genotyped. Locus MSTA133 was excluded from the analyses due to high variation in the number of repetitive dinucleotide motifs, resulting in alleles with a size over 300 bp being unable to assemble correctly. Both diploid individuals (GILL, CYG) and their seed progeny were fully homozygous across the studied loci (Table 4), as was expected due to the prevailing autogamy in these two species. However, due to low coverage, it was not possible to determine the alleles of the MSTA53 locus in CYG and its seed progeny. The triploid taxa (CRI, PUD) had one to three alleles per locus. Notably, locus MSTA78 in PUD showed only two alleles of comparable dosage, suggesting the presence of a null allele. All tested progeny possessed identical genotypes with their maternal individuals, as expected from the mode of reproduction (autogamy in diploid and obligate apomixis in triploid taxa; Table 4). The results confirm the capacity of SSR-seq to genotype seeds of the apomictic complex from the *Asteraceae family*. The alignment of each locus and detected alleles among the studied *Taraxacum* species can be found in Supporting Information Figure S2.

**Table 4:** SSR genotypes of the studied *Taraxacum* individuals and their seed progeny (TRX set), including the ploidy levels of embryos and endosperms based on FCSS

Species	Maternal individual ID	Seed ID	Ploidy emb:end	MSTA53	MSTA78	MSTA131
***T. cygnorum***	**CYG**			**Low coverage**	**B4**	**C5**
*T. cygnorum*	CYG	Cyg-1	2:3	Low coverage	B4	C5
*T. cygnorum*	CYG	Cyg-2	2:3	Low coverage	B4	C5
*T. cygnorum*	CYG	Cyg-3	2:3	Low coverage	B4	C5
*T. cygnorum*	CYG	Cyg-4	2:3	Low coverage	B4	C5
*T. cygnorum*	CYG	Cyg-5	2:3	Low coverage	B4	C5
***T. cristatum***	**Ga5**			**A3**	**B3b, B3d, B3e**	**C1, C2, C5**
*T. cristatum*	Ga5	Ga5-1	3:6	A3	B3b, B3d, B3e	C1, C2, C5
*T. cristatum*	Ga5	Ga5-2	3:6	A3	B3b, B3d, B3e	C1, C2, C5
*T. cristatum*	Ga5	Ga5-4	3:6	A3	B3b, B3d, B3e	C1, C2, C5
*T. cristatum*	Ga5	Ga5-6	3:6	A3	B3b, B3d, B3e	C1, C2, C5
*T. cristatum*	Ga5	Ga5-w1	3:6	A3	B3b, B3d, B3e	C1, C2, C5
*T. cristatum*	Ga5	Ga5-w2	3:6	A3	B3b, B3d, B3e	C1, C2, C5
*T. cristatum*	Ga5	Ga5-w3	3:6	A3	B3b, B3d, B3e	C1, C2, C5
*T. cristatum*	Ga5	Ga5-w4	3:6	A3	B3b, B3d, B3e	C1, C2, C5
***T. gilliesii***	**GILL**			**A1**	**B1**	**C6**
*T. gilliesii*	GILL	Gill-1–1	2:3	A1	B1	C6
*T. gilliesii*	GILL	Gill-1–2	2:3	A1	B1	C6
*T. gilliesii*	GILL	Gill-3	2:3	A1	B1	C6
*T. gilliesii*	GILL	Gill-4	2:3	A1	B1	C6
*T. gilliesii*	GILL	Gill-5	2:3	A1	B1	C6
***T. pudicum***	**Pud25/F1**			**A2, A2, A4**	**B2, B3g, null**	**C1, C3, C4**
*T. pudicum*	Pud25/F1	Pud25-1	3:6	A2, A2, A4	B2, B3g, null	C1, C3, C4
*T. pudicum*	Pud25/F1	Pud25-2	3:6	A2, A2, A4	B2, B3g, null	C1, C3, C4
*T. pudicum*	Pud25/F1	Pud25-3	3:6	A2, A2, A4	B2, B3g, null	C1, C3, C4
*T. pudicum*	Pud25/F1	Pud25-4	3:6	A2, A2, A4	B2, B3g, null	C1, C3, C4
*T. pudicum*	Pud25/F1	Pud25-6	3:6	A2, A2, A4	B2, B3g, null	C1, C3, C4
*T. pudicum*	Pud25/F1	Pud25-w1	3:6	A2, A2, A4	B2, B3g, null	C1, C3, C4
*T. pudicum*	Pud25/F1	Pud25-w2	3:6	A2, A2, A4	B2, B3g, null	C1, C3, C4
*T. pudicum*	Pud25/F1	Pud25-w3	3:6	A2, A2, A4	B2, B3g, null	C1, C3, C4
*T. pudicum*	Pud25/F1	Pud25-w4	3:6	A2, A2, A4	B2, B3g, null	C1, C3, C4

Bold indicates maternal individual and its genotype

SSR-seq analysis provided insights into the allelic composition of the parental individuals and seed progeny. However, for the diploid *Rubus ulmifolius* (RJV5), it was not possible to amplify loci 53E02 and 1B06 (Table 5). In all investigated tetraploid individuals, locus 1B06 amplified more alleles than expected from the ploidy level. Based on the sequence variability, it was possible to discern two potential paralogues, with one amplifying in all parental individuals and displaying greater individual heterozygosity. Only this one was accepted in further analyses. Locus RiM015 was excluded due to the length of the alleles over 300 bp being unable to assemble correctly. The alignment of each locus and detected alleles among the studied *Rubus* taxa can be found in Supporting Information Figure S3.

**Table 5:** SSR genotypes of the parental *Rubus* individuals, including the ploidy levels and dosage of each allele based on the sequencing coverages

Species	Individual ID	Ploidy level	Rub47a	FruitC1	RhM023	72H02	Rub238h	53E02	1B06
*R. apricus*	MS176/20	4	A8,A8,A12,A15	B1,B1,B2,B2	C2,C3,C3,C3	D1,D1,D1,D5	G1,G1,G5,G5	H2,H5,H8,H11	I5,I5,I10,I13
*R. bifrons*	R150-20	4	A1,A3,A5,A8	B2,B4,B5,B7	C1,C1,C2,C3	D1,D1,D2,D3	G2,G3,G3,G5	H1,H4,H6,H6	I1,I2,I4,I6
*R. epipsilos*	R11-10	4	A4,A5,A5,A8	B1,B4,B5,B6	C1,C3,C3,C3	D1,D1,D1,D2	G3,G4,G5,G5	H6,H6,H7,H8	I1,I4,I6,I8
*R*. ser. *Glandulosi*	R143-9	4	A2,A6,A6,A8	B1,B3,B4,B8	C3,C3,C3,C3	D1,D1,D1,D1	G1,G2,G2,G5	H2,H3,H5,H8	I3,I3,I8,I8
*R.* ser. *Glandulosi*	Kornik	4	A3,A8,A13,A13	B1,B2,B4,B12	C3,C3,C5,C6	D1,D1,D1,D7	G5,G5,G5,G5	H2,H3,H7,H8	I3,I4,I8,I10
*R.* ser. *Glandulosi*	MS137/20	4	A3,A6,A6,A8	B2,B3,B12,B12	C1,C3,C3,C3	D1,D1,D1,D1	G2,G5,G5,G5	H2,H7,H7,H17	I3,I4,I8,I10
*R.* ser. *Glandulosi*	MS165/20	4	A3,A8,A8,A11	B1,B4,B4,B8	C5,C5,C6,C7	D1,D1,D1,D7	G5,G5,G5,G5	H1,H7,H10,H16	I3,I4,I10,I10
*R.* ser. *Glandulosi*	MS196/20	4	A3,A3,A13	B1,B1,B4,B12	C1,C3,C3,C7	D1,D1,D1,D7	G5,G5,G5,G5	H2,H3,H7,H8	I4,I4,I4,I10
*R. ulmifolius*	RJV5	2	A3,A3b	B2,B2	C1,C1	D2,D3	G1,G3	null	null
*R. vatavensis*	R127-12	4	A5,A6,A7,A8	B5,B7,B8,B8	C2,C3,C3,C3	D1,D1,D1,D1	G2,G2,G5,G5	H1,H1,H6,H6	I1,I4,I5,I5
*R. vatavensis*	R127-4	4	A5,A6,A7,A8	B2,B5,B7,B8	C1,C2,C3,C3	D1,D1,D1,D2	G2,G2,G5,G5	H1,H1,H6,H6	I1,I4,I5,I6

For loci abbreviations, see Supporting Information Table S2

Based on the sequencing coverage, the allelic dosage could be accurately determined in all polyploid parental individuals of *Rubus* (Table 5) and most of the embryos. Exact determination of the dosage was not possible only in some seed progenies, as is visible from repetitions of SSR-seq of several seeds (Table 6, Supporting Information Tables S6 and S7). This limitation was mostly related to the low allelic richness of the locus, where a maximum of two alleles per individual were observed. Thus, careful interpretation of allele dosage is needed for loci exhibiting low inter-individual variability, specifically those with a number of alleles equal to or less than half the ploidy level.

**Table 6:**
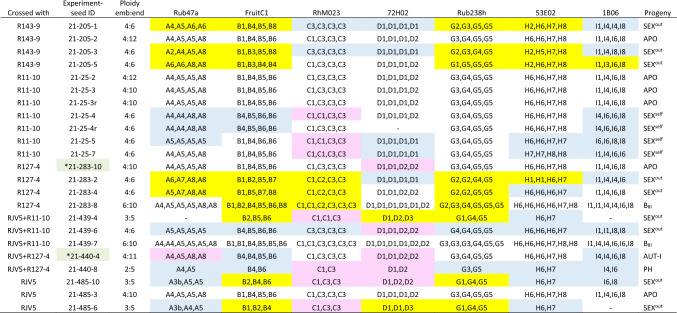
SSR genotypes of seed progeny of *R. epipsilos* (individual R11-10), including the ploidy levels of embryos and endosperms based on FCSS.

**r** – repetition; green* genotyping differing from expected based on FCSS mode reproduction determination. APO – apomictic, SEX^out^ – sexual out-crossing, SEX^self^ – sexual selfing, AUT-I – automixis type I, AUT-II – automixis type II (for explanation of automixis see Fig. 1), PH – polyhaploid, B^III^ – hybrid with elevated ploidy. Coloured boxes mark progeny genotypes differing from the maternal individual: yellow – an extra allele, blue – a missing allele, purple – changed dosage. For SSR genotypes of parental individuals, see Table 5; for SSR genotypes of all *Rubus* progeny, see Supporting Information Tables S6 (RUB*ex* set) and S7 (RUB*nat* set)

In the *Rubus* seed progeny that originated from recombination of two parental genomes (RUB*ex* set, crossing experiment types ii and iii; FCSS embryo:endosperm = 2C:3C), SSR-seq genotyping confirmed the presence of maternal and paternal alleles with comparable sequencing coverages in all cases, with minor variations in dosage/presence of alleles in maximum one locus per individual seed. Considering all seven loci in the analysis, the Polygene program successfully discerned the true paternal individuals within the tetraploid offspring of the RUB*ex* set, as delineated in Supporting Table S8. When a reduced number of loci (2–6) were employed, misassignments manifested in a maximum of two seeds out of the thirteen (plus two repetitions). Notably, one of the seeds was autogamous, resulting in reduced heterozygosity. Consequently, combining data from all seven loci proved sufficient for determining the paternal individual among the studied *Rubus* species.

Furthermore, in all four seeds from hybridization experiment iii (stigma pollinated with pollen mixture from variable donors, i.e., ALL; Supporting Information Table S6) that had parthenogenetic embryos, it was possible to determine the pollen donor participating in fertilization of the central cell (endosperm formation; data not provided). The presence of paternal alleles in the endosperm was significantly lower compared to the number of embryonic cells. Thus, exact determination of paternal individuals was not possible for progeny where the pollen donor was unknown (RUB*nat* set). The main reason in such cases is the difficulty distinguishing real sequence variability (i.e., paternal alleles in endosperm) from PCR/sequencing errors. However, with sufficient coverage and a known genotypic pool in the population, the pollen donor can be identified for apomictic seeds based on the genotype of the endosperm. Unless specified otherwise, all presented results are based only on genotyping the embryos, ignoring low-coverage contigs.

Most of the parthenogenetic embryos of tetraploid *Rubus* (FCSS embryo:endosperm ≤ 2C:5C) had the maternal genotype. However, contrary to expectation, sixteen seeds of suggested apomictic origin did not possess all maternal alleles in the exact dosage as the maternal individual (Table 6, Supporting Information Tables S6 and S7). Among these seeds, nine showed altered dosage in a single locus, two lacked one allele in a single locus, and two exhibited a novel allele at a single locus. These observed differences might have arisen from mutations resulting in novel or null alleles or incorrect determination of allelic dosage in loci with a maximum of two alleles per tetraploid. Additionally, the genotypes of three seeds showed variations in multiple loci. Two of these seeds originated from the crossing experiment (seed IDs: 21–440-4 and 21–238-4), where both parents were known (RUB*ex* set; Table 6, Supporting Information Table S6), and one seed (seed ID: MS137/20–3) was identified in the RUB*nat* set (Supporting Information Table S6). Automixis type I could explain the genotypes observed in these progeny.

Based on the combination of FCSS and SSR-seq results, it was also possible to estimate the levels of autogamy and allogamy in natural populations (RUB*nat* set). Progeny was considered to arise from selfing if FCSS suggested sexual origin (embryo:endosperm ratio 2C:3C) and the embryo's genotype lacked some of the maternal alleles or exhibited altered dosage in more than one locus while not acquiring novel alleles. This assumption was confirmed by all three sexually originated seeds from selfing in crossing experiments (RUB*ex* set treatment (i). Among the 26 sexually derived seeds analysed in the RUB*nat* set, sixteen were determined to originate from autogamy, and nine originated from allogamy (Supporting Information Table S7). Nonetheless, selfing cannot be distinguished from outcrossing with genetically similar individuals or from automixis type II. Given that automixis type II was not detected in the RUB*ex* set, the explanation of lost alleles due to this type of reproduction is doubtful.

The variability of embryo ploidy levels suggested by FCSS analyses in the investigated tetraploid *Rubus* was also confirmed by SSR genotyping (Table 6, Supporting Information Tables S6 and S7). When FCSS analysis suggested parthenogenetic development of reduced egg cells (polyhaploid formation), SSR-seq confirmed this reproduction mode, with only half of the maternal alleles recovered within diploid embryos. Conversely, when embryos with increased ploidy levels (hexaploidy) were identified based on the FCSS analysis suggesting B_III_ hybrid formation, SSR-seq revealed all maternal alleles enriched by the paternal alleles from the pollen donor.

The progeny of *Rubus* resulting from both experiments were categorized based on the combination of FCSS and SSR-seq results, reflecting their origin (Table 3). The representation of each category in each maternal individual is summarized in Table 7.

**Table 7:** The representation of variable embryo origin based on the combination of FCSS and SSR-seq.

Set	Species	Maternal individual	APO	SEX^out^	SEX^Self^	AUT-I	AUT-II	PH	B_III_
RUB*ex*	*R. bifrons*	R150-20	16	1	–	1	–	2	2
	*R. epipsilos*	R11-10	5	9	3	1	–	1	2
	*R. vatavensis*	R127-12	5	1	–	–	–	–	1
	*R. vatavensis*	R127-4	1	2	–	–	–	2	–
	*R*. ser. *Glandulosi*	R143-9	11	–	–	–	–	–	2
RUB*nat*	*R*. ser. *Glandulosi*	Kornik	4	1	2	–	–	2	1
	*R*. ser. *Glandulosi*	MS137/20	-	–	9	1	–	–	–
	*R*. ser. *Glandulosi*	MS165/20	5	1	2	–	–	–	2
	*R*. ser. *Glandulosi*	MS196/20	-	7	3	–	–	–	–
	*R. apricus*	MS176/20	8	–	–	–	–	2	–

RUB*ex* – maternal individuals and seed progeny of *Rubus* from experimental crosses, RUB*nat* – maternal individuals and seed progeny of *Rubus* collected in nature. APO – apomictic, SEX^out^ – sexual out-crossing, SEX^self^ – sexual selfing, AUT-I – automixis type I, AUT-II – automixis type II (for explanation of automixis see Fig. 1), PH – polyhaploid, B_III_ – hybrid with elevated ploidy.

## Discussion

The combination of FCSS and SSR-seq has great potential for accurately assessing the mating system of flowering plants. In our study, we applied both methods to investigate the mode of reproduction in two enigmatic genera, *Taraxacum* and *Rubus*, and concluded that while FCSS is helpful for rapid screening, it possesses some limitations (see also Dobeš et al. 2013). Similarly, SSR-seq alone cannot clearly distinguish between different mating systems, such as polyhaploidy and selfing, or B_III_ hybrids and normal sexual progeny. Both approaches may thus lead to an overestimation of a certain type of reproductive mode if used separately.

One of the limitations of FCSS stems from the possibility of endosperm formation from a single unreduced polar nucleus resulting in the same FCSS ratio as for sexual reproduction, known, for example, in some species of Panicoideae (Warmke 1954; Kaushal et al. 2018). This anomaly should also be taken into consideration in the genus *Rubus*, where the retardation of polar nuclei fusion before endosperm development has been observed and hypothesized to be primarily associated with the apomictic mode of reproduction (Czapik 1983, 1985b). However, our results did not confirm this hypothesis, as all RUB*ex* seeds with a 2C:3C FCSS ratio confirmed their sexual origin through SSR-seq data. Similarly, Dobeš et al. (2013) failed to prove endosperm formation from a single polar nucleus in *Potentilla*, although they could not rule it out completely in the series *Tomentillae*. However, further research should consider expanding the sample size by including more seeds and genetic loci or exploring various apomictic taxa. The methodology presented in this study holds promise for uncovering additional reproductive mechanisms.

The FCSS method may be further limited by the potential occurrence of automixis. This reproductive mode has been previously identified in *Rubus caesius* based on cytoembryology, where a reduced megagametophyte was observed, and diploid chromosome number was restored through the fusion of two haploid nuclei produced by the division of an egg nucleus (Gerlach 1965). Such ploidy restoration would result in full homozygosity in the progeny, which was not observed in our datasets. Automixis was also suggested to explain minisatellite fingerprints of artificial hybrids in diploid *R. idaeus* and the tetraploid blackberry cultivar 'Majestät' (Antonius and Nybom 1995). Based on crossing experiments, cytological observations, and available literature, Dowrick (1961, 1966) even proposed that conventionally understood apomixis is not an essential reproductive mechanism in tetraploid brambles. According to those works, apomictic progeny are produced through diploidization of the reduced egg cell by restitution during its first division or by fusion with another nucleus in the embryo sac. This would have significant implications for the validity of the FCSS results, as both sexually and apomictically/automictically derived seeds would display the 2C:3C FCSS ratio. However, unreduced megagametophytes are often detected in polyploid *Rubus* taxa, contradicting Dowrick's conclusions. In our experimental crossing, all seeds with an embryo:endosperm genome size ratio of 2C:3C carried alleles of both parents, thus originating through the combination of two parental genotypes.

All the abovementioned studies considered type II automixis (Fig. 1d), where somatic chromosome number is restored in the reduced megagametophyte, similar to gamete duplication in parthenogenetic animals (Mirzaghaderi and Hörandl 2016). However, in theory, restitution can also be achieved through the fusion of megaspores (automixis type I; Fig. 1c), which would resemble the terminal or central fusion of reduced nuclei in parthenogenetic animals (Cook 1993). The consequences of type I automixis differ from type II in the rate of decreasing heterozygosity in progeny and the FCSS profile, as the embryo sac is unreduced, resulting in a 2C:5C FCSS ratio typical for apomixis. Two seeds from our RUB*ex* and one from RUB*nat* sets exhibited this ratio, and at the same time, their embryonic SSR genotype differed from the maternal genotype by missing alleles and changing dosages in more than a single locus. Additionally, no paternal alleles were detected in sufficient dosages to be identified as embryonic, although they were detected in low dosages forming endosperm. The most plausible explanation for this pattern is type I automixis, although, to our knowledge, it has not been previously observed in angiosperms. These three automictically derived seeds accounted for 2.59% of our dataset and 5.77% of the progeny with unreduced embryo sacs, suggesting that automixis may not be an infrequent event in facultative apomicts. To determine the frequency of automixis in the reproductive systems of apomicts, it is necessary to assess the reproductive mode in a robust number of progeny and loci.

## Conclusions

The presented approach combines FCSS and SSR-seq methods at the single-seed level. The analysis of *Rubus* demonstrated the usefulness of this approach in validating FCSS results by genotyping each progeny seed and the capacity to detect automixis. It is applicable in variable seed sizes, as shown on the offspring of *Taraxacum,* and we successfully tested the approach in other systems (*Potentilla* and *Hieracium)*. Furthermore, this method shows great potential for directly quantifying autogamy levels in natural populations. It is applicable not only to sexual plants or sexual progeny of facultative apomicts but also to apomictic progeny in pseudogamous taxa through endosperm genotyping, eliminating the need for challenging seed germination in some instances. It also addresses concerns about the reliability of the FCSS method in taxa with deviated reproductive pathways, such as the nonstandard fusion of nuclei in megagametophytes. Additionally, SSR-seq analysis of the seeds—a method that has never been used hitherto—may serve as an attractive alternative to FCSS in angiosperm taxa where FCSS cannot differentiate between apomictic and sexually derived progeny (e.g., apomictic grasses). SSR-seq offers advantages in situations where FCSS faces challenges due to the presence of secondary compounds interfering with DNA staining (Jedrzejczyk and Sliwinska 2010), the occurrence of G2 phase peaks or endopolyploidy (Krahulcová and Rotreklová, 2010), or difficulties in detecting endosperm peaks with a low number of endosperm nuclei (Dobeš et al. 2013).

## Supplementary Information

Below is the link to the electronic supplementary material.

Supplementary file1 (PDF 1195 kb)

Supplementary file2 (XLSX 18 kb)

Supplementary file3 (XLSX 17 kb)

Supplementary file4 (XLSX 102 kb)

## Data Availability

The datasets generated and analysed during the current study are available from the corresponding author upon request.
