# Supplementary material for: A novel strategy to study apomixis, automixis, and autogamy in plants
Source: Plant Reprod. 2024 Mar 2;37(3):379–92. doi: 10.1007/s00497-024-00499-6 (PMC11377528; doi:10.1007/s00497-024-00499-6)
Supplement: Supplementary file 1 — Supplementary file1 (PDF 1195 kb) [file 497_2024_499_MOESM1_ESM.pdf]

Article title: A novel strategy to study apomixis, automixis, and autogamy in plants.

Journal name: Plant Reproduction

Authors: Petra Šarhanová, Ľuboš Majeský, Michal Sochor

Corresponding author: Petra Šarhanová, E-mail: sarhanova@gmail.com, Masaryk University, Department of Botany and Zoology, Kamenice 753/5, 625 00 Brno, Czech Republic

The following Supporting Information is available for this article:

Fig. S1 Examples of coverage graphs of *Rubus* loci

Fig. S2 Alignments of detected alleles of *Taraxacum* loci

Fig. S3 Alignments of detected alleles of *Rubus* loci

Fig. S4 Histograms from flow cytometric seed screen

Table S1 List of *Taraxacum* loci

Table S2 List of *Rubus* loci

Table S3 PCR conditions

Table S4 The origin of embryo sacs and embryos in *Taraxacum*

Table S5 The origin of embryo sacs and embryos in *Rubus*

Table S6 Genotypes and ploidy levels of parental individuals and progenies in RUB*ex* set

Table S7 Genotypes and ploidy levels of maternal individuals and progenies in RUB*nat* set

Table S8 Parentage analyses in the program POLYGENE

Fig. S1 Examples of selected coverage graphs of Rubus loci. Every graph shows a single individual. Read coverage of contigs is scaled to 1. The red line separates true alleles (above) and false alleles (below) originating from sequencing/PCR errors or endosperm. For loci abbreviations, see Supporting Information Table S2; for the ID of each individual, see Table 1

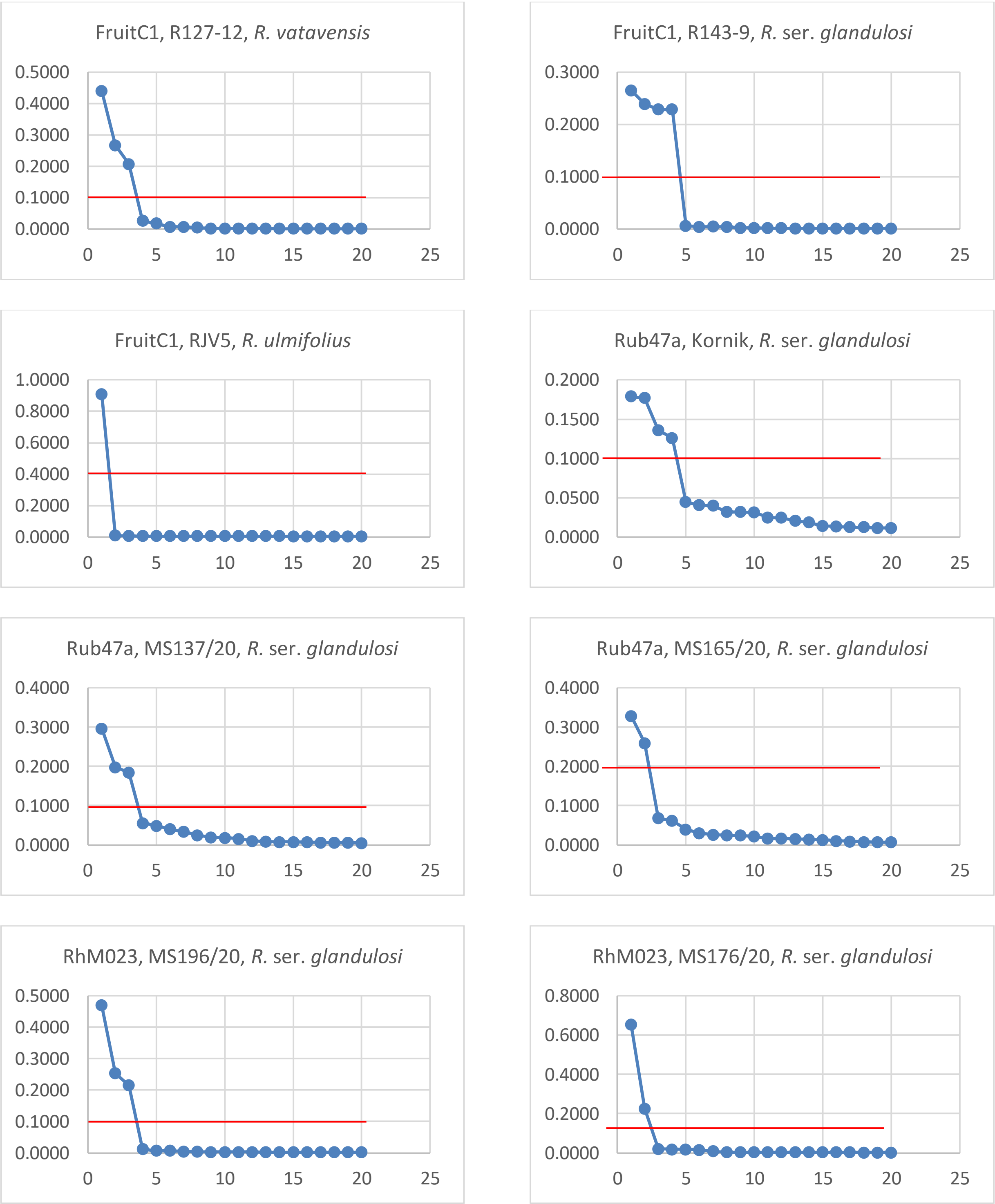

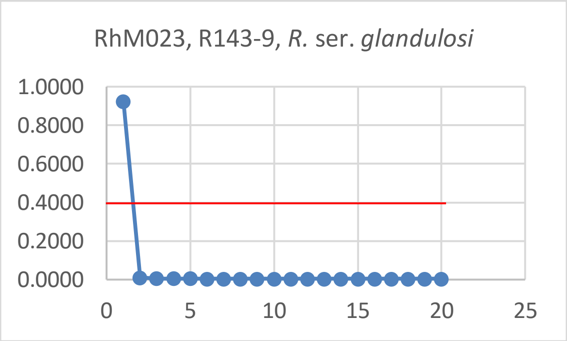
RhM023, R143-9, *R.* ser. *glandulosi*
1.0000
0.8000
0.6000
0.4000
0.2000
0.0000
0 5 10 15 20 25

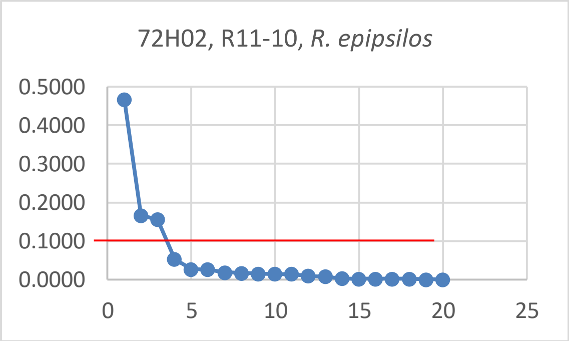
72H02, R11-10, *R. epipsilos*
0.5000
0.4000
0.3000
0.2000
0.1000
0.0000
0 5 10 15 20 25

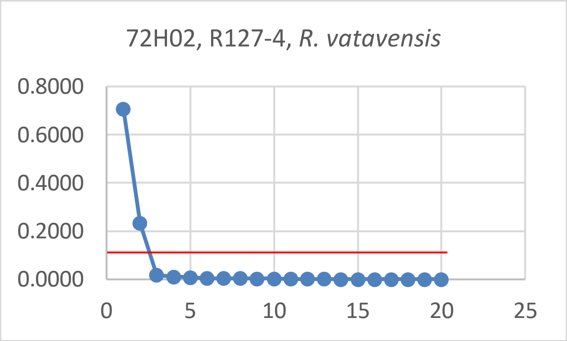
72H02, R127-4, *R. vatavensis*
0.8000
0.6000
0.4000
0.2000
0.0000
0 5 10 15 20 25

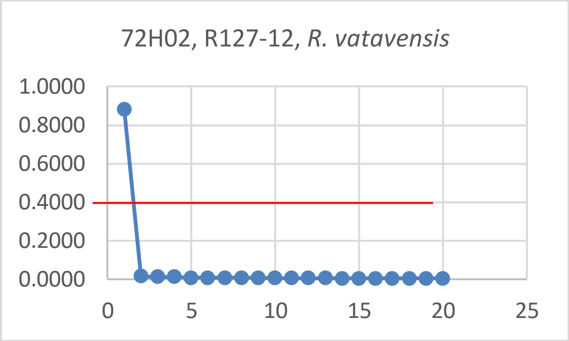
72H02, R127-12, *R. vatavensis*
1.0000
0.8000
0.6000
0.4000
0.2000
0.0000
0 5 10 15 20 25

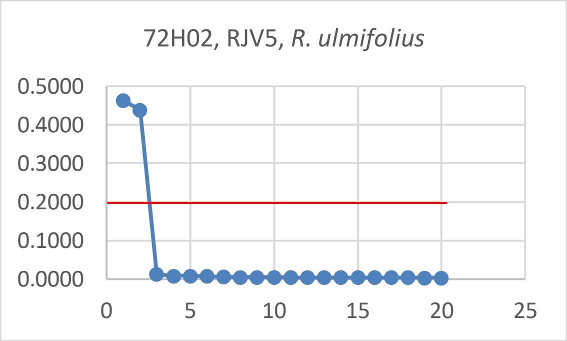
72H02, RJV5, *R. ulmifolius*
0.5000
0.4000
0.3000
0.2000
0.1000
0.0000
0 5 10 15 20 25

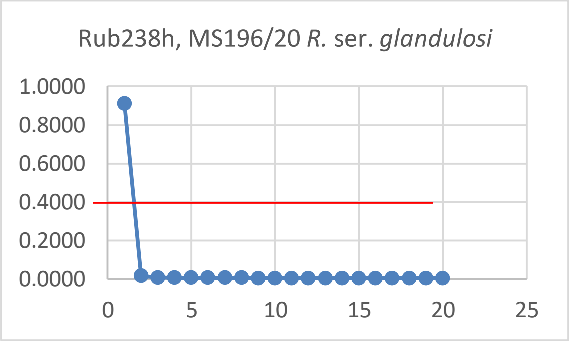
Rub238h, MS196/20 *R.* ser. *glandulosi*
1.0000
0.8000
0.6000
0.4000
0.2000
0.0000
0 5 10 15 20 25

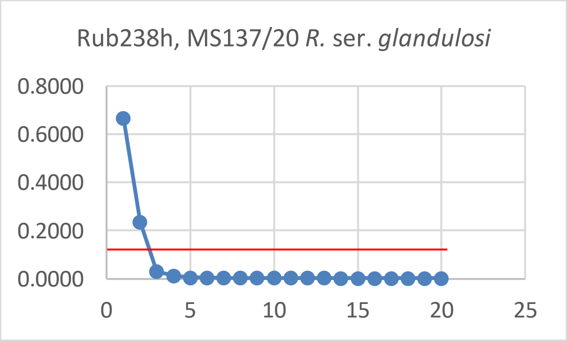
Rub238h, MS137/20 *R.* ser. *glandulosi*
0.8000
0.6000
0.4000
0.2000
0.0000
0 5 10 15 20 25

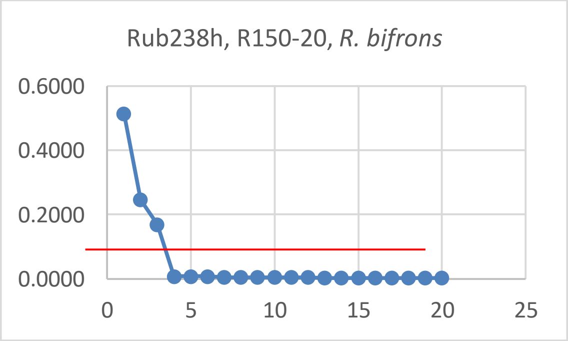
Rub238h, R150-20, *R. bifrons*
0.6000
0.4000
0.2000
0.0000
0 5 10 15 20 25

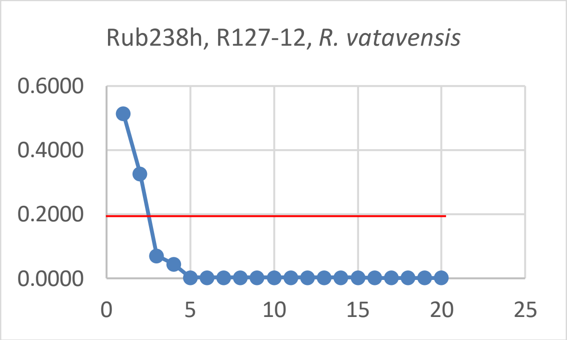
Rub238h, R127-12, *R. vatavensis*
0.6000
0.4000
0.2000
0.0000
0 5 10 15 20 25

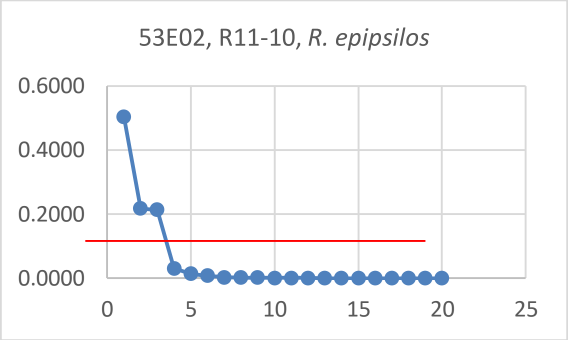
53E02, R11-10, *R. epipsilos*
0.6000
0.4000
0.2000
0.0000
0 5 10 15 20 25

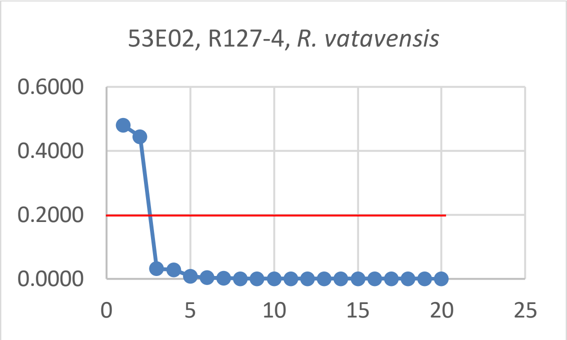
53E02, R127-4, *R. vatavensis*
0.6000
0.4000
0.2000
0.0000
0 5 10 15 20 25

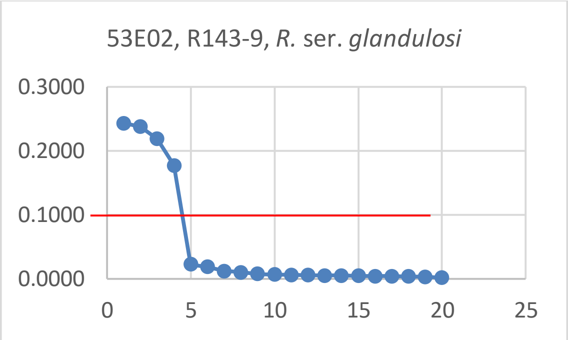
53E02, R143-9, *R.* ser. *glandulosi*
0.3000
0.2000
0.1000
0.0000
0 5 10 15 20 25

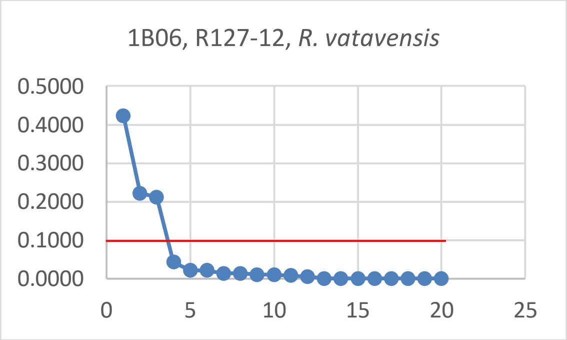
1B06, R127-12, *R. vatavensis*
0.5000
0.4000
0.3000
0.2000
0.1000
0.0000
0 5 10 15 20 25

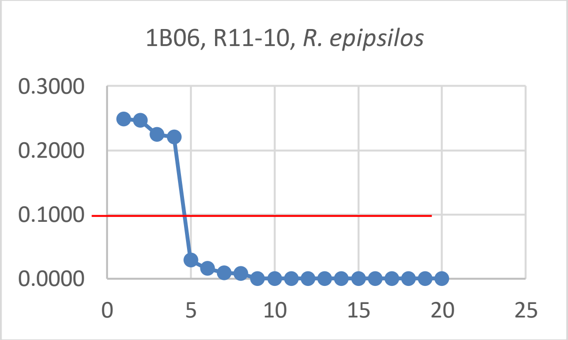
1B06, R11-10, *R. epipsilos*
0.3000
0.2000
0.1000
0.0000
0 5 10 15 20 25

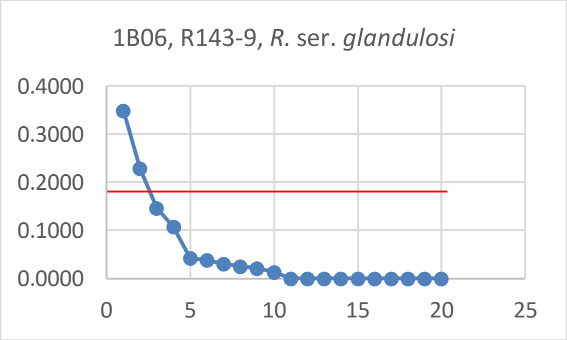
1B06, R143-9, *R.* ser. *glandulosi*
0.4000
0.3000
0.2000
0.1000
0.0000
0 5 10 15 20 25

Fig. S2 Alignments of detected alleles in studied *Taraxacum* species and loci MSTA53, MSTA78, and MSTA131.

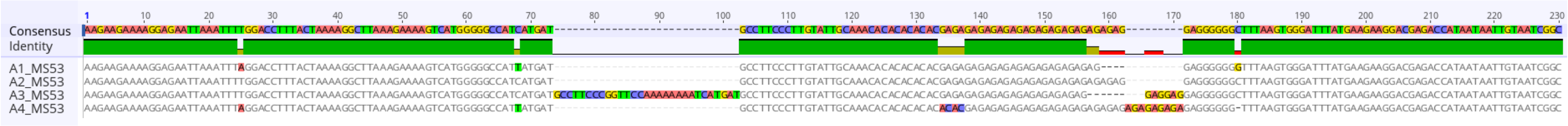

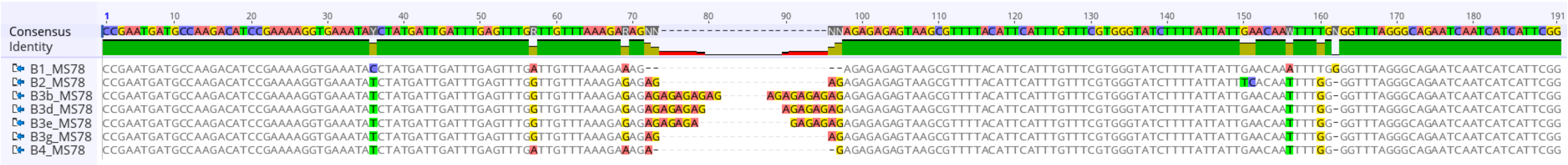

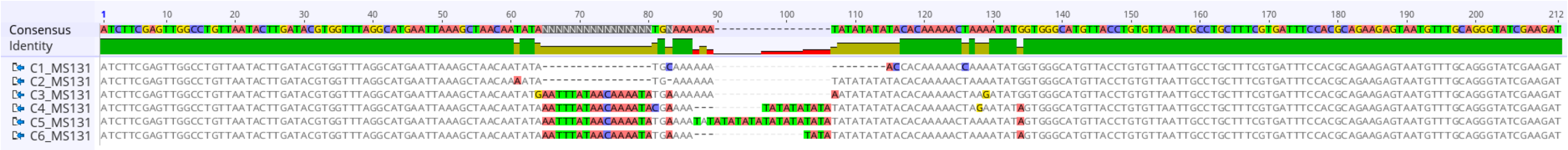

Fig. S3 Alignments of detected alleles in studied *Rubus* species and loci Rub47a, FruitC1, RhM023, 72H02, Rub238h, 53E02, 1B06. For loci abbreviations, see Supporting Information Table S2.

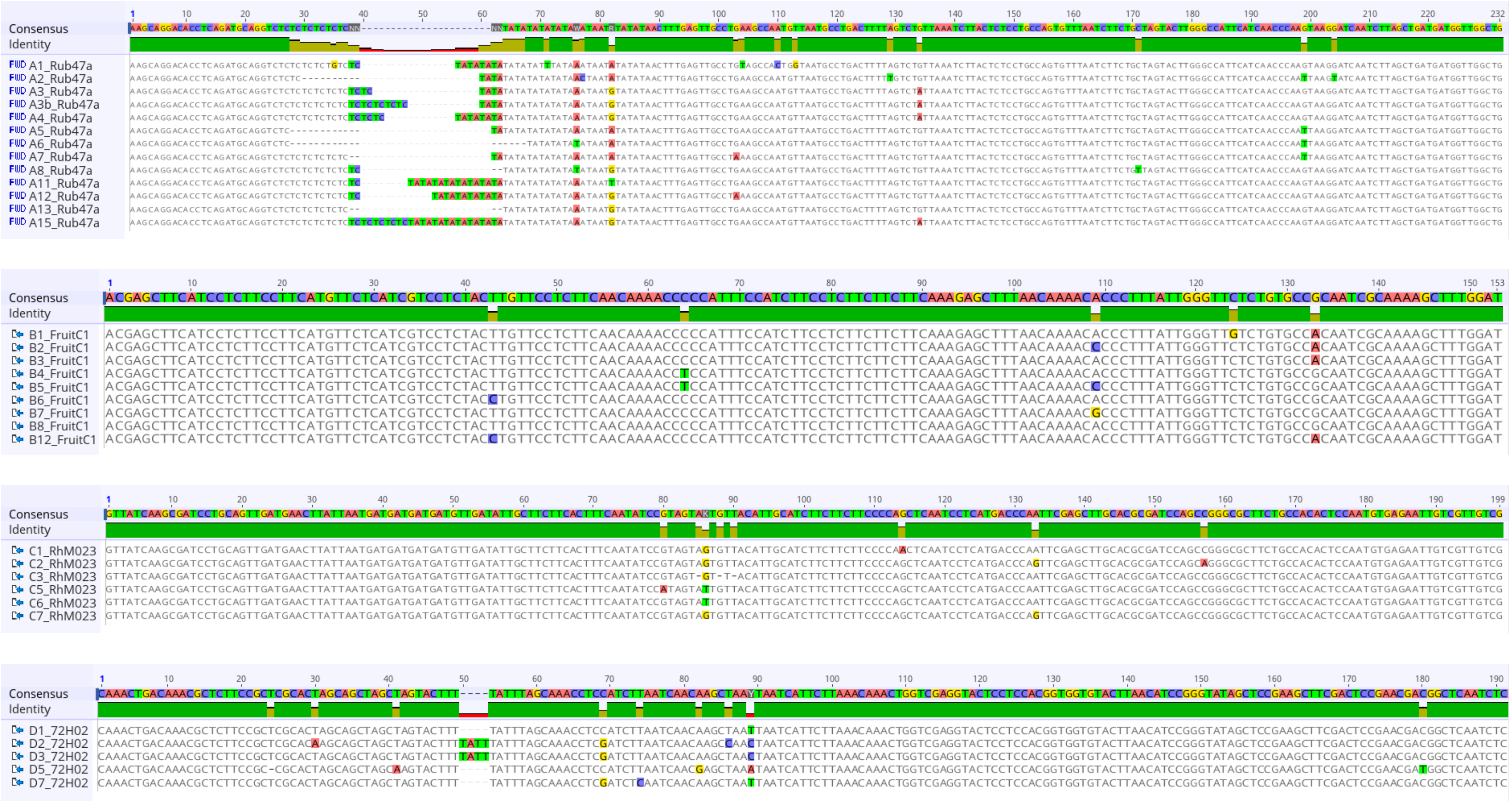

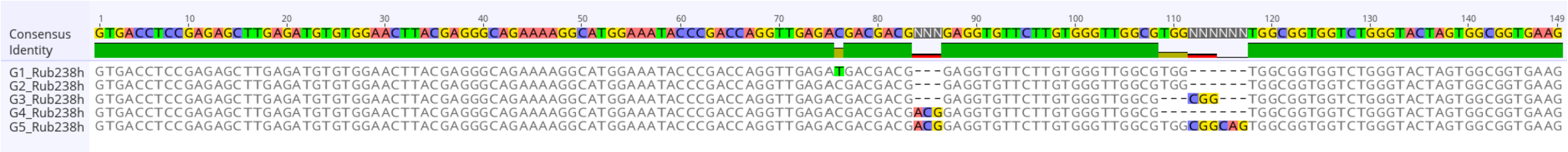

Consensus
Identity
G1_Rub238h
G2_Rub238h
G3_Rub238h
G4_Rub238h
G5_Rub238h

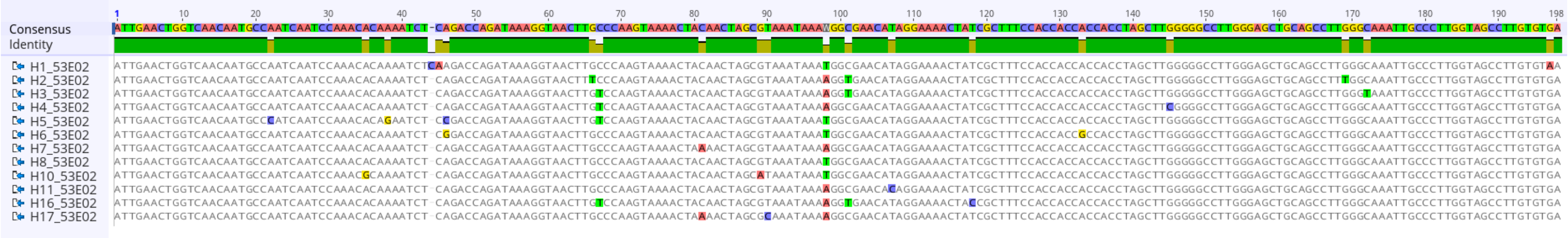

Consensus
Identity
H1_53E02
H2_53E02
H3_53E02
H4_53E02
H5_53E02
H6_53E02
H7_53E02
H8_53E02
H10_53E02
H11_53E02
H16_53E02
H17_53E02

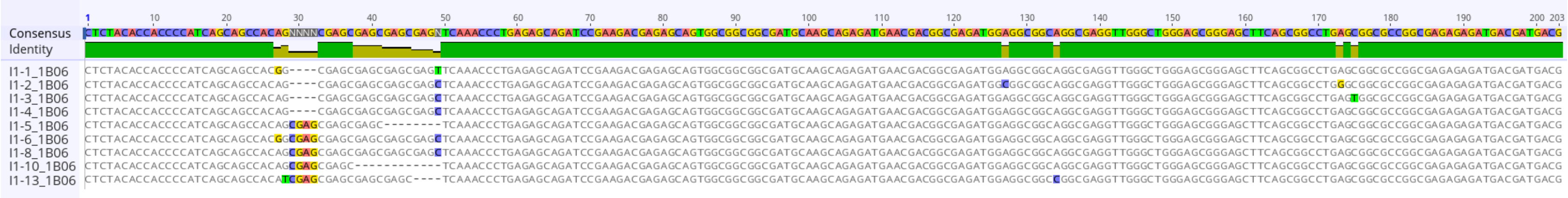

Consensus
Identity
I1-1_1B06
I1-2_1B06
I1-3_1B06
I1-4_1B06
I1-5_1B06
I1-6_1B06
I1-8_1B06
I1-10_1B06
I1-13_1B06

Fig. S4 Examples of histograms from flow cytometry seed screen of *Taraxacum* and *Rubus* seeds. x-axis – relative fluorescence; y-axis – number of particles. a) diploid and b) triploid *Taraxacum* seeds; c) diploid, d) triploid, e-g) tetraploid and h) hexaploid *Rubus* seeds with variable endosperm ploidy levels.

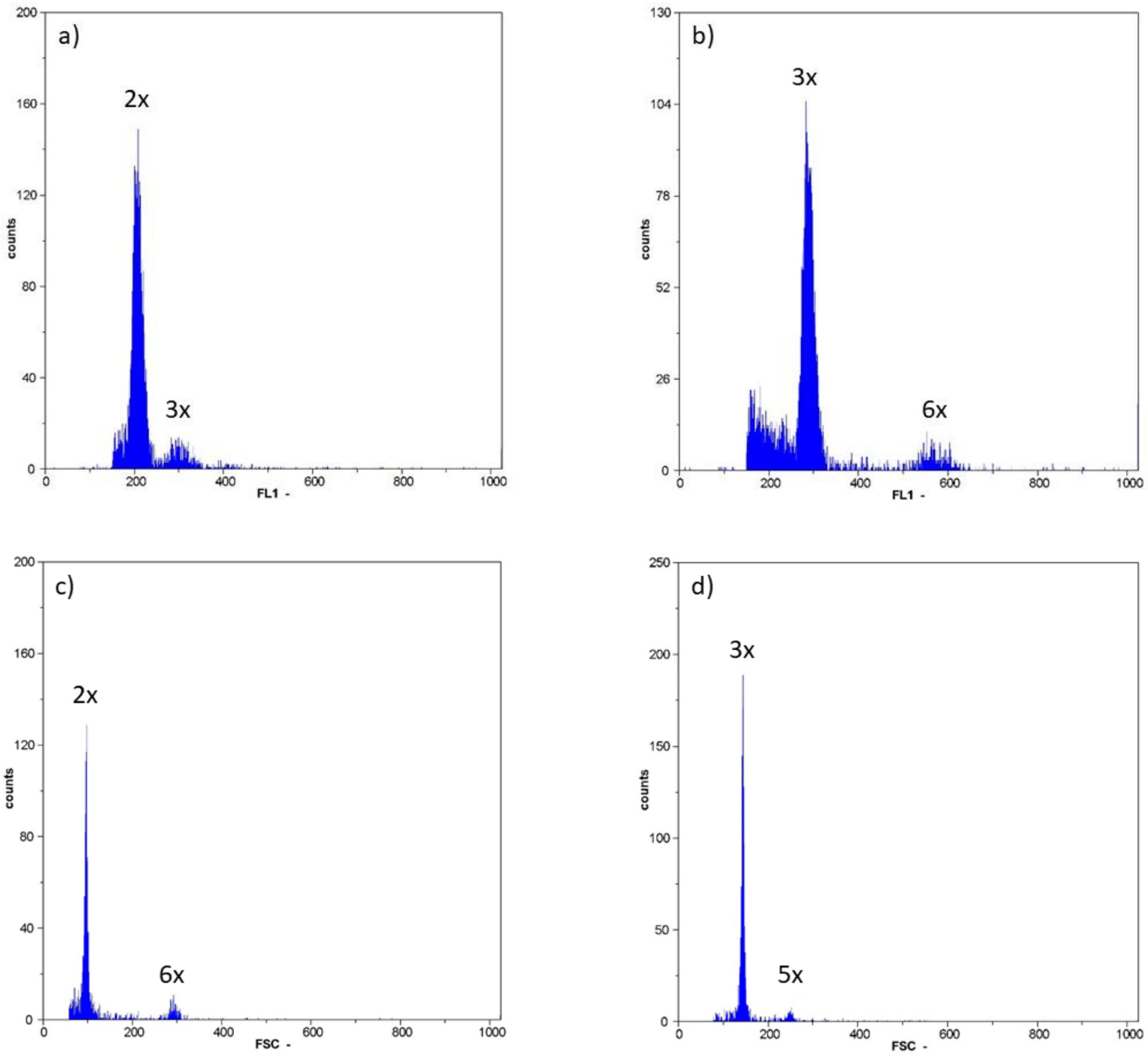

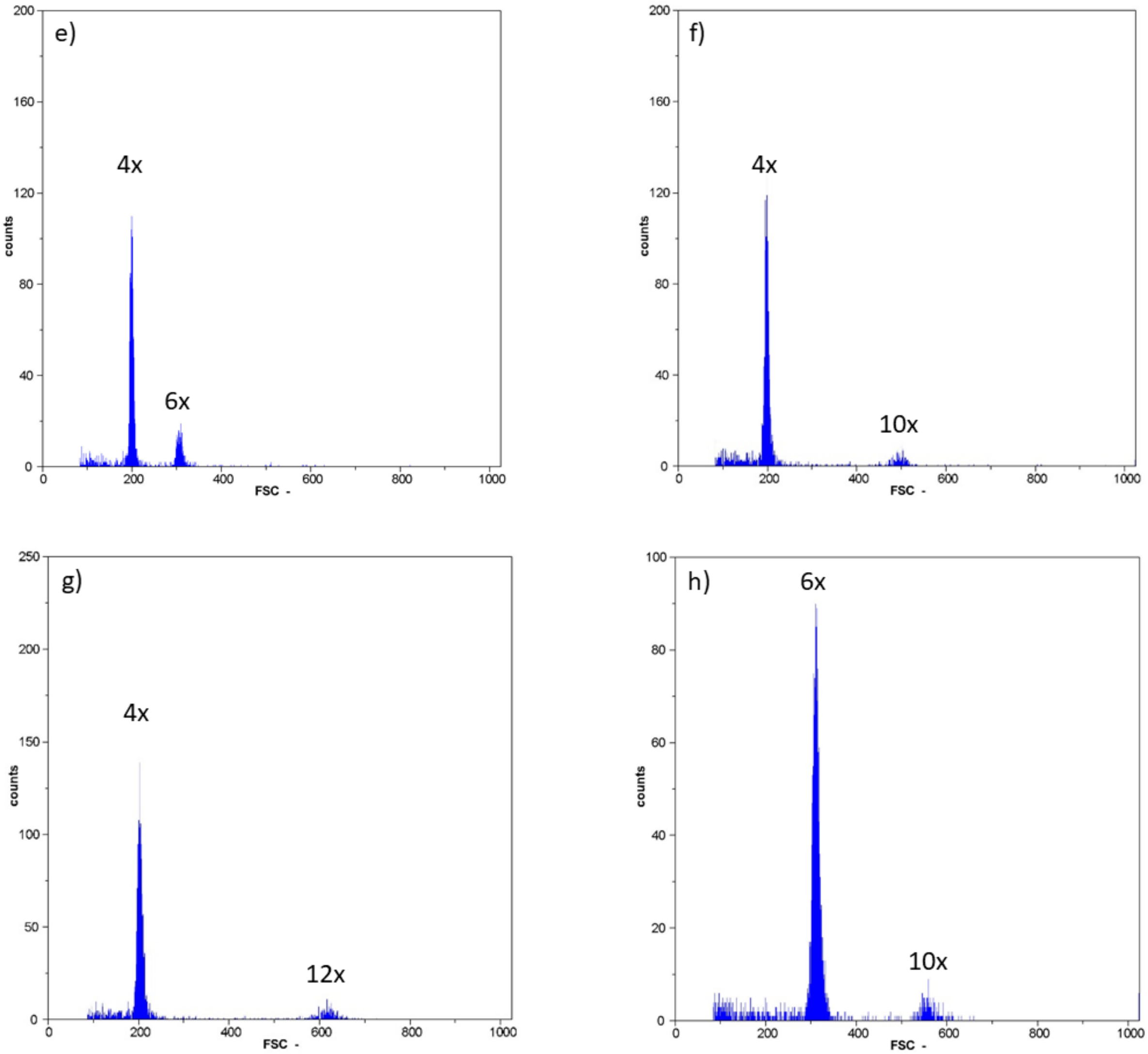
e)
4x
6x
counts
FSC -
0
40
80
120
160
200
0
200
400
600
800
1000
f)
4x
10x
counts
FSC -
g)
4x
12x
counts
FSC -
0
50
100
150
200
250
h)
6x
10x
counts
FSC -
0
20
40
60
80
100

Table S1 List of tested SSR loci for *Taraxacum* with detected sizes and SSR motifs. Loci MSTA44B and MSTA93 did not amplify in all parental species and were excluded from analyses.

Locus	Length	SSR motif	Forward primer	Reverse primer	Reference
MSTA44B	179-187	GA	AGTTTCTCTAAAATGGGAAGAT	TGTCAGGTATATTCAAAAGATTC	Falque *et al.*, 1998
MSTA53	188-230	AC+GA	CAATTATTATGGTCTCGTCCTT	CCAGTTGAAGCAAAAACAGT	Falque *et al.*, 1998
MSTA78	165-190	GA	TGATTGATTCTGCCCTAAACC	TGCCAAGACATCCGAAAAG	Falque *et al.*, 1998
MSTA93	239-253	TC	GTTTTGTTTGGGTTTGGTATTGTC	ATGCCCCCTCTATGTCCTAT	Vašut *et al.*, 2004
MSTA131	171-214	TA	TACCCTGCAAACATTACTCTTCTG	GTTGGCCTGTTAATACTTGATACG	Vašut *et al.*, 2004
MSTA133	>300	CA+CT+GA	CATATGACCCACGACAAAATCTAA	GCTGCCCTATGCCCACTGC	Vašut *et al.*, 2004

Table S2 List of tested SSR loci for *Rubus* with detected sizes and SSR motifs. Loci RH_MEa0005aH07, ERubLR_SQ9.4_B05, and RubPara_SQ007_O09 did not amplify in all parental species and were excluded from the analyses. Locus ERubLR_SQ8.2_D06 did not amplify in any parental species. Detected length and SSR motif are provided. LG – linkage group.

Locus	Abbreviation	LG	Length	SSR motif	Forward primer	Reverse primer	Reference
ERubLR_SQ01_B06	1B06	4	191-203	AGCG	CTCTACACCACCCCATCAG	CGTCATCGTCATCTCTCTCG	Woodhead *et al.*, 2008; Šarhanová *et al.*, 2017
Rub238h	Rub238h	3	142, 151	ACG+TGG	GTGACCTCCGAGAGCTTGAG	CTTCACCGCCACTAGTACCC	Woodhead *et al.*, 2008; Šarhanová *et al.*, 2017
Rubusr47a	Rub47a	1	202-220	TC+TA	AAGCAGGACACCTCAGATGC	CAGCCAACCATCATCAGCTA	Graham *et al.*, 2004; Šarhanová *et al.*, 2017
RhM023	RhM023	?	196, 199	ACT+CAT	CGACAACGACAATTCTCACATT	GTTATCAAGCGATCCTGCAGTT	Castillo & Reed, 2010
ERubLR_SQ07_2_H02	72H02	4	234, 238	TAAA	TGGCAATCAACCACTCTGTG	CAAACTGACAAACGCTCTTCC	Woodhead *et al.*, 2008
RubLR_SQ05_3_E02	53E02	5	197, 198	GGT	TCACACAAGGCTACCAAG	ATTGAACTGGTCAACAATGC	Woodhead *et al.*, 2008
RubfruitC1	FruitC1	2	169	CTT	ACGAGCTTCATCCTCTTCC	ATCCAAAGCTTTTGCGATTG	Graham *et al.*, 2004
RiM015	RiM015	3	>300	TCA	CGACACCGATCAGAGCTAATTC	ATAGTTGCATTGGCAGGCTTAT	Castillo & Reed, 2010
RH_MEa0005aH07	5aH07	4	197-209	TCA+TA	CTGGTCTGATGGTGGGACTG	AGTGTGCCTACCTCCACTCT	Castro *et al*. 2013
ERubLR_SQ9.4_B05	94B05	6	193	TGG	GAGCATGTTATTAAGCCTGTTATCAA	TAGGGTCCTTTCCAGAGAACG	Woodhead *et al*. 2013
RubPara_SQ007_O09	7O09	2	291-295	CAT	CATGGAAAACCATGCATCATA	CTTTGTCCAAAAGTGCTGT	Woodhead *et al.*, 2008
ERubLR_SQ8.2_D06	82D06	6	-	-	TGGCTGAGGCTTACACCAAG	AGGAAGGAAGCTAGGAAATAGAGA	Woodhead *et al*. 2013

Table S3 Conditions and grouping of loci in multiplex PCR reactions. The concentration of each primers stock solution is 10µM; RUB1 – first multiplex of *Rubus* loci, RUB2 – second multiplex of *Rubus* loci, TRX – multiplex of *Taraxacum* loci, MM – master mix.

RUB1	µl
MM	5.0
ERubLR_SQ01_B06_F	0.3
ERubLR_SQ01_B06_R	0.3
Rub238h_F	0.3
Rub238h_R	0.3
Rubusr47a_F	0.2
Rubusr47a_R	0.2
RhM023_F	0.15
RhM023_R	0.15
H2O	2.1
DNA	1.0

RUB2	µl
MM	5.0
ERubLR_SQ07_2_H02_F	0.3
ERubLR_SQ07_2_H02_R	0.3
RubLR_SQ05_3_E02_F	0.15
RubLR_SQ05_3_E02_R	0.15
RubfruitC1_F	0.2
RubfruitC1_R	0.2
H2O	2.7
DNA	1.0

TRX	µl
MM	5.0
MSTA53_F	0.5
MSTA53_R	0.5
MSTA78_F	0.2
MSTA78_R	0.2
MSTA131_F	0.2
MSTA131_R	0.2
H2O	1.2
DNA	1.0

Table S4 The number of reduced and unreduced embryo sacs and embryos originating from fertilization or parthenogenetic development of tested *Taraxacum* individuals.

Species	ID	reduced fertilized	reduced parthenogenetic	unreduced fertlized	unreduced parthenogenetic
T. gilliesii	GILL	10	0	0	0
T. cygnorum	CYG	10	0	0	0
T. pudicum	PUD 25	0	0	0	10
T. cristatum	GA5	0	0	0	10

Table S5 The number of reduced and unreduced embryo sacs and embryos originating from fertilization or parthenogenetic development of tested tetraploid *Rubus* individuals.

Species	ID	reduced fertilized	reduced parthenogenetic	unreduced fertilized	unreduced parthenogenetic
R. bifrons	R150-20	1	2	2	15
R. epipsilos	R11-10	12	1	2	6
R. vatavensis	R127-12	1	0	1	5
R. vatavensis	R127-4	2	2	0	1
R. ser. *Glandulosi*	R143-9	0	0	2	11
R. ser. *Glandulosi*	Kornik	3	2	0	5
R. ser. *Glandulosi*	MS137/20	9	0	0	1
R. ser. *Glandulosi*	MS165/20	3	0	2	5
R. ser. *Glandulosi*	MS196/20	10	0	0	0
R. apricus	MS176/20	0	2	0	8

Table S6 SSR-genotypes of the studied *Rubus* parental individuals used for the crossing experiment (RUB*ex* set) and seed progeny, including the ploidy levels of embryos and endosperms based on FCSS. r – repetition; green* genotyping differing from expected based on FCSS mode reproduction determination. APO – apomictic, SEX^{out} – sexual out-crossing, SEX^{self} – sexual selfing, AUT-I – automixis type I, AUT-II – automixis type II (for the explanation of automixis see Fig. 1), PH – polyhaploid, B_{III} – hybrid with elevated ploidy. Colored boxes mark progeny genotype differing from the maternal individual: yellow – extra allele, blue – missing allele, purple – changed dosage.

Table S7 SSR-genotypes of the studied Rubus maternal individuals and their progeny collected in nature (RUB*nat* set, including the ploidy levels of embryos and endosperms based on FCSS. r – repetition; green* genotyping differing from expected based on FCSS mode reproduction determination. APO – apomictic, SEX^{out} – sexual out-crossing, SEX^{self} – sexual selfing, AUT-I – automixis type I, AUT-II – automixis type II (for the explanation of automixis see Fig. 1), PH – polyhaploid, B_{III} – hybrid with elevated ploidy. Colored boxes mark progeny genotype differing from the maternal individual: yellow – extra allele, blue – missing allele, purple – changed dosage.

Table S8 Determination of the offspring's parentage in POLYGENE of tetraploid sexually originated seeds of *Rubus* from crossing experiment RUB*ex* set based on the LOD score (the natural logarithm of likelihoods that the individual is and is not the paternal). The individual with the highest positive LOD score is considered the true paternal individual. r – repetition.
